# Diabetes, pregnancy and spina bifida

**DOI:** 10.25122/jml-2021-0249

**Published:** 2021

**Authors:** Tiberiu Georgescu, Olivia Ionescu, Paris Ionescu, Nicolae Bacalbasa, Lucian Gheorge Pop

**Affiliations:** 1. National Institute of Mother and Child Care Alessandrescu-Rusescu, Bucharest, Romania; 2. Department of Obstetrics and Gynecology, Carol Davila University of Medicine and Pharmacy, Bucharest, Romania; 3. Department of Obstetrics and Gynecology, South Nurnberg Hospital, Nurnberg, Germany; 4. Department of Obstetrics and Gynecology, Ovidius University, Constanta, Romania

**Keywords:** spina bifida, diabetes, ultrasound, neural tube defects

## Abstract

Spina bifida is a disorder characterized by failure of the neural tube to form during embryological development. The early signs in the head and spine may be detected on ultrasound from 11 weeks of gestation. Diabetes is a well-known teratogen factor that increases the chances of birth defects, such as neural tube defects. We report a 12 weeks case of spina bifida in type 1 diabetes.

A nuchal translucency scan is a standard requirement for assessing fetal anatomy. First-trimester diagnosis of spina bifida can be a daunting task. It requires an understanding of the abnormal pathophysiological process, knowledge of patients’ history, ultrasound signs, and good scanning images, which is a prerequisite for a correct diagnosis. Recent advances in scanning technology have enabled us to diagnose spina bifida in the first trimester with greater accuracy and confidence. Here, we report a case of a 24-year woman, GII PI, with type 1 diabetes who was referred to our clinic for first-trimester screening at 12 weeks plus four days of gestation. This was an unplanned pregnancy, and her pre pregnancy blood sugar levels were poorly controlled, above ten mmol/l. Transabdominal and transvaginal ultrasounds were performed. The dorsal-anterior and dorsoposterior midsagittal views of the spine highlighted the open spinal defect in the lumbosacral region of the spine referred to as myeloschisis (Figure 1). Brain stem/Brain stem occipital bone ratio is inverted (Figure 2). After proper counseling of both parents, the couple opted for pregnancy termination. Upon macroscopic examination, the most striking morphological defect was the presence of an open spinal dysraphism in the lumbosacral region. Histologically, the sac was lined by skin, dura proper, and arachnoidal cells (Figures 3 and 4). As the neural tube closes at day 28–32 of gestation, even before many women are aware of their pregnancy, we believe it is of utmost importance that folic acid intake before conception should be a routine practice in any woman that envisages a pregnancy.

**Figure 1.**
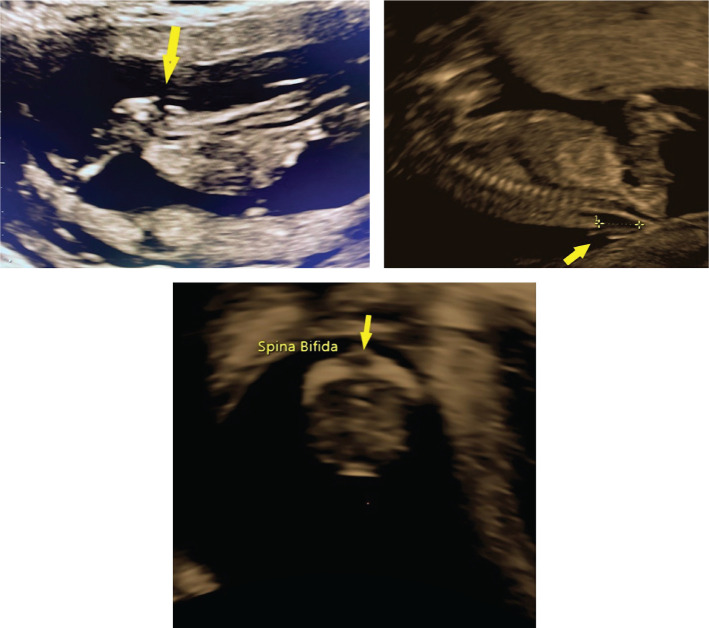
Top left: Transabdominal 2D midsagittal view of the spine showing the defect in the lumbosacral region. Top right: Corresponding transvaginal 2D midsagittal view of the defect referred to as myeloschisis. Bottom: Multiplanar reconstruction-axial view-plan B illustrating the spine protruding through the lumbosacral spine

**Figure 2.**
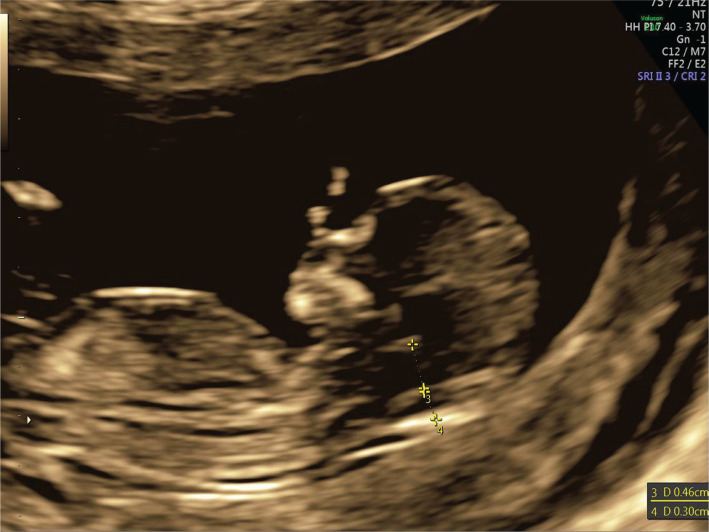
Inverted Brain Stem/Brain Stem Occipital Bone ratio.

**Figure 3.**
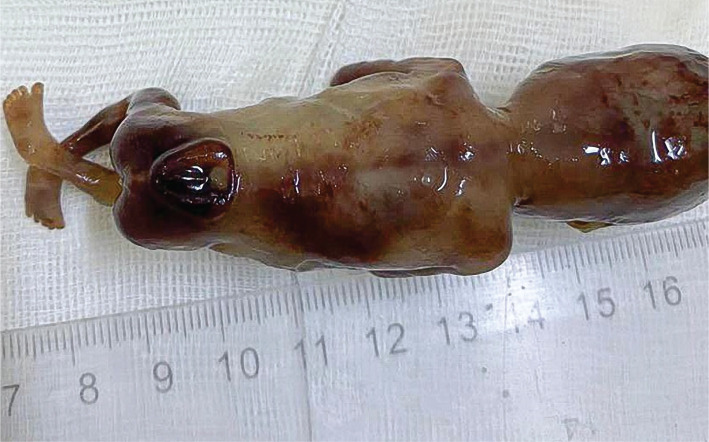
The specimen of the fetus showing the protrusion of the spine in the lower lumbosacral region.

**Figure 4.**
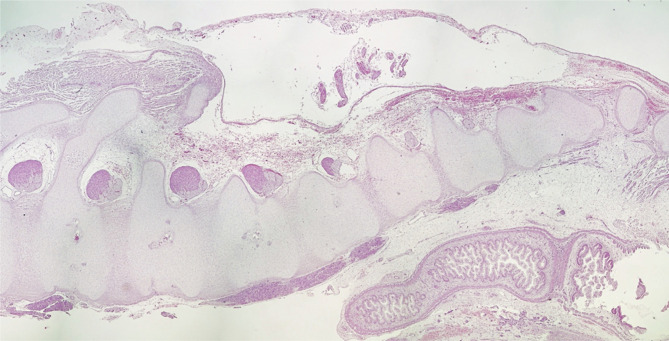
Stitched images showing a histopathological aspect of the spinal dysraphism in the lumbosacral region in the lumbosacral region (Hematoxylin and eosin, 15x).

## Consent to publish

Informed consent to publish the data was obtained from the participant.

